# Labor linkages and flow paths of industry in China

**DOI:** 10.1016/j.heliyon.2024.e30118

**Published:** 2024-04-26

**Authors:** Xuan Li, Yueyang Li, Yu Song

**Affiliations:** a The Institute for Sustainable Development, Macau University of Science and Technology, Macau, 999078, China; b Business School, Macau University of Science and Technology, Macau, 999078, China

**Keywords:** Input-output models, Labor force population, Industry linkage effects, Improved hypothetical extraction method, Structural path analysis

## Abstract

As economic power increases and market patterns adapt, labor becomes an increasingly significant factor of production. However, there is a dearth of discourse regarding the structural changes that have occurred in the correlation of the labor force across industries, as well as a visual representation of the labor force's movement across industries. To quantify and analyze the correlation effect with greater precision, it is necessary to establish an input-output model as the foundation of analysis, comparing the changes in the total output of the economic system prior to and subsequent to the exclusion using the vertical integration algorithm. By decomposing the path structure, the average propagation distance of the labor force population's demand for each industry can be determined. By employing labor force population data from the corresponding years and China's input-output tables published by the National Bureau of Statistics of China (NBS) from 2005 to 2020, this study conducts a quantitative analysis of the correlation effect between labor force population and the trend of its transfer across 19 industries. The findings indicate that the correlation effect between labor force and population is most pronounced in the manufacturing sector. Furthermore, the construction sector faces an especially critical requirement for labor force personnel from other industries. The article culminates with a recommendation that the government enhance its macro-control endeavors to address labor market risk shocks and take an active stance in response to labor market fluctuations.

## 1 Introduction

Many scholars have conducted numerous empirical studies on the labor force population in China, focusing on analyzing the spatial structure of labor mobility. Scholars have employed methods like fractal theory, Electronics System Design Automation (ESDA), hotspot analysis, spatial regression, Moran's index, and geographically weighted regression model to illustrate population mobility patterns at different administrative levels, but there are still constraints. Common comprehensive evaluation methods include principal component analysis [1], hierarchical analysis process [2], multi-criteria decision analysis [3], grey assessment methods [4], factor analysis [5] and fuzzy integrated assessment [6]. Each integrated assessment method has its advantages, but they are mainly suitable for interregional studies. Some methods prioritize qualitative components over quantitative data, potentially resulting in subjective and less persuasive outcomes. A new integrated model needs to be developed that is closely linked with input-output analysis to thoroughly examine the shifts in labor force mobility among industries.

Achieving full employment for the labor force is a fundamental objective of China's economic and social reform and development, serving as a critical juncture for the government as it formulates a multitude of economic and social policies-as a populous and developing nation [7]. In recent years, as a result of the adjustment of market patterns and the expansion of economic strength, the cost of production in China has risen gradually, as has the size of the labor force population, and the composition of the labor force population has evolved. Hence, it is imperative to investigate the pertinent ramifications of labor force utilization across various industries in China [[8], [9], [10]]. Concurrently, employed individuals encountered obstacles in resuming their work, job hunting became more challenging, and the living service industry, small and micro enterprises and self-employed businessmen were profoundly affected [11]. Conversely, the magnitude of the 2019 pandemic's influence on workforce participation across sectors exhibits significant variation. Specifically, the accommodation and catering industry and the construction industry have relatively low resumption rates by the end of February 2020 (43 % and 40 %, respectively). Late in March, the percentage of workers awaiting the resumption of work remained at around 20 %, and the working population in these sectors faces a high risk of unemployment [12].

A thorough examination of the urban survey unemployment rate, the demand multiplier, and the inflation rate, among other things, has enabled the majority of scholars to explicate the labor market transmission channels in terms of supply and demand [13]. In this particular context, it is critical to quantify the significance of the labor force population and its vertically integrated consumption.

The present study investigates the effects of industrial labor on the population. In doing so, it departs from the prior research framework that relied on intuitive economic indicators and instead capitalizes on the flawless predictability and stability of input-output models [14]. The hypothetical extraction method (HEM) is first proposed by Schultz, which assumes that a sector is extracted out of the economic system. By comparing the total output change of the economic system before and after the sector extraction, we can determine the impact of the sector on economic system [15]. HEM, which analyzes data structured on the basis of sectoral linkages and input–output models [16], has become one of the most widely used empirical research paradigms in trade association research [17]. Conventional approaches to examining correlation effects place greater emphasis on intermediate inputs [18]. Consequently, to achieve a more precise and quantitative analysis of the correlation effect, the modified hypothetical extraction method (MHEM) integrates the HEM with vertically integrated measures (VIM). This integration enables the classification of the correlation effect into four distinct factors: internal effect, composite effect [19], net prophase shift and net post phase shift [20].

Nevertheless, the existing scholarly investigation concerning the correlation effect in China's labor population by industry is not comprehensive in nature and has yet to tackle the intricate correlation effect that pertains to labor population transfer between industries. Therefore, it is necessary to consider the correlation effect of the labor population in each industry in China, the total direct and indirect labor population in each industry, and the four factors of quantitative analysis of the correlation effect of the labor population in the National Bureau of Statistic's four-year input–output table from 2015 to 2020.

Hypothesis extraction research, which examines the degree of association of a particular factor among diverse industries in a given region, has emerged as a prominent area of interest in international economics from a theoretical standpoint [21]. APL also refers to the average number of steps required to calculate the reverse demand pull (or positive supply push) of one industry that influences the output of another industry. Theoretical reference significance abounds in relation to its application in China's labor population market analysis, employment guidance, and policy formulation concerning employment.

## 2 Literature review

Recently, scholars have started examining the connections among factors like factor mobility, industrial changes, and the regional economy. Some of the literature evaluates how carbon emissions affect economic growth in various sectors. Certain researchers employ a spatial panel econometric model to study how regional industrial shifts influence carbon emissions at the provincial level [22]. The literature has increasingly incorporated regional economic linkages, carbon transfer, and industrial transfer-induced labor mobility into the same research framework as investigations have advanced. As one of the most significant factors of production, labor spatial allocation influences all facets of society and the economy through its impact on industry inputs, outputs, trade, and consumption. China, being a developing and populous nation, places significant emphasis on attaining full employment of its labor force as a primary objective of its economic and social development reforms. This objective serves as a crucial foundation for the government as it devises a multitude of economic and social policies [23]. Nevertheless, there is a higher frequency of industrial transfers between regions that share closer economic ties. These transfers invariably result in fluctuations in employment demand within each region. Therefore, industrial agglomeration and industrial transfers jointly dictate the movement of labor factors, specifically the trajectory of their movement. Labor mobility subsequently influences the trend and trajectory of industrial transfers. Shifts in labor mobility, demand, and availability result in industry transformations [24]. Interregional input-output modeling, which connects regional input-output models, is utilized to reflect economic ties between industries within regions as part of this trend [25]. The examination of labor mobility via industrial transfer offers an additional lens through which to analyze energy or pollution issues derived from industrial studies. Thus, studies pertaining to energy or pollution, including carbon emissions, may find some utility in the application of input-output models for labor mobility measurement.

A comprehensive examination of the urban survey unemployment rate and the job search multiplier (amount of jobs/number of job seekers) has led to the conclusions of several academics. Proposing time-sensitive and convenient income support programs, they advised the government to increase employment and stimulate consumption in the short term. An exhaustive examination incorporating the demand multiplier, the survey unemployment rate, and the core inflation rate indicates that the present unemployment rate has acquired a discernibly cyclical nature, yet this does not fundamentally affect the labor market's economic stability [26]. On the contrary, in the event of labor market instability, the consequence will be an inadequate labor supply, industry shutdowns, and the discontinuation of service sector consumer services [27]. With labor demographic market shocks, such as industrial shutdowns and the cessation of consumer services, a full-blown recession eventually occurs [28]. Therefore, it is essential to conduct an in-depth analysis of the labor force population's effects on various industries in China, as well as the labor force population's effects that are industry specific. Fang Li determined, via an examination of supply and demand data, that the labor force population has transitioned from primary and secondary industries to the tertiary sector, with an indication that this trend will continue to gain momentum over time [29].

The linkage effect and the transfer of the labor population force between industries have not yet been thoroughly investigated in academic studies on the use of the labor population force in China's various industries. Furthermore, investigations pertaining to the correlation of the labor force population predominantly encompass various metrics such as the unemployment rate, demand multiplier, and inflation rate, among others. Different industries in China are difficult to discern the effects of the labor force population, and the labor force population has an industrial correlation effect. An area that currently lacks in scholarly investigation is the systematic examination of the linkage effect and the transfer of the labor force population between industries in China. Furthermore, a more comprehensive analysis of the linkage effect and its implications for industry utilization remains unfinished. HEM must be used to measure the direct and indirect use of the labor force population in different industries in China in recent years, quantify and analyze the four factors of labor force population-related effects, and investigate the transfer of the labor population force between industries in China, including the transfer paths and average transmission distance of the transfer paths.

Analytically examining the rule of value flow, internal correlation, quantity dependence, and supply–demand equilibrium among diverse sectors of the national economy is the purpose of an input–output model. Wassily Leontief, a professor and 1973 Nobel Laureate in economics, initially introduced and elaborated upon the concept of input–output. HEM compares the changes in the total output of the economic system before and after the exclusion of a particular industry in order to determine the industry's correlation effect with other industries and its significance to the entire economic system. The input–output model serves as the analytical foundation for HEM [30]. HEM has become one of the more popular empirical research paradigms in industrial linkage studies and is used in many fields [31,32]. HEM has been applied not only to analyze the impact of sectoral structural changes on the economy, but also in the areas of resources [33], energy and environmental protection [34]. Presently, alongside the traditional approach, the virtual elimination method has gained significant traction as a paradigm for empirical investigations concerning industry linkages. By employing assumptions to “eliminate” the linkage effects of specific industries within the general framework of the input–output model, the approach aims to assess the linkage effects between industries through a comparison of the changes in the overall economic system's output prior to and subsequent to elimination. HEM can decompose sectoral linkages by separating a sector from the economic system; furthermore, it is more precise than the conventional approach, which has contributed to its increased adoption in recent research [35]. The modified hypothetical extraction method (MHEM), which combines HEM with longitudinal integration algorithms and divides the correlation effect into four factors-internal effect, composite effect, net forward, and net backward transfer [34] has been widely implemented in the study of related field effects and enables more precise quantification and analysis of the correlation effect [[36], [37], [38]].

This study presents a model that examines the labor-population correlation effect of individual industries within the framework of MHEM. It conducts an analysis of the intersectoral transfer trend of labor-population correlation effect in cross-sectoral comparisons and employs Dietzenbacher's structural path analysis (SPA) method to quantify the amplification effect resulting from the nonlinear propagation of influence along sectoral correlation paths via structural decomposition of the path mu. By structurally decomposing path multipliers, the method quantifies the amplification effect caused by nonlinearities in the propagation of impacts along sectoral paths. By quantifying the amplification effects resulting from nonlinearities in the propagation of impacts along sector-linked paths via the structural decomposition of path multipliers and the average propagation distance (APL) required for the labor force population to increase, on average, the final demand for the outputs of one sector to influence the demand of another sector, the methodology identifies intersectoral shifts in particular industries [39,40]. In summary, the MHEM-based analysis of the four factors of labor population force correlation effects and labor population force transfer between industries has unique advantages in the study of this issue [41].

## 3 Research methodology

Research methodology is a tool and instrument that we must use to carry out academic research, and the following research methodology has been adopted in this paper.

### 3.1 Input-output model

The Input-output Model encompasses a variety of structural data integrated into the System of National Accounts (SNA), along with theories from different schools of thought and methodologies based on structural data. One major benefit of input-output tables is the access to data on intermediate inputs derived from the national accounting framework, which is a key factor contributing to their extensive use by researchers. The input-output table is characterized by a rigorous and transparent data framework, as well as a self-contained and logical mechanism. The measurement of parameters in the input-output model differs significantly from other methods. It relies on data from national economic accounting and various specialized surveys. The parameter estimation process follows strict guidelines within the national economic accounting system, ensuring a rigorous and transparent approach compared to subjective historical methods [42,43].

Input-output tables facilitate technological analysis by providing a detailed description of the inputs needed for the production activities of a sector. Input-output analysis examines how technological changes in certain sectors impact the economy by analyzing how the output of one sector influences the input of another sector [44].

### 3.2 Hypothetical extraction method

The idea of HEM was initially captured by Schultz first proposed the HEM and used it to investigate the impact of changes in a single industry on the economic system in which it is located. The importance of an industry to the whole economic system and the correlation effect between different industries are analyzed [45]. Nowadays, HEM has become a popular empirical research paradigm in industrial association research and is used in many fields.

HEM estimates the loss of the total gross output after hypothetically extracting a productive sector the empirical literature uses both TLM and HEM liberally to estimate interindustry linkages, but there appears to be a preference for HEM because it reflects more sensitively the interindustry relationships existing in the economies [46]. Assume that the economic system β consists of n industries, $X_{\text{ij}}$ denotes the inputs of industry i to j, $y_{i}$ denotes the final demand of industry i, the final demand vector of industry i is denoted as $Y=\left(y_{i}\right)$, the output vector of industry i is denoted as $X=\left(x_{i}\right)$, and $x_{j}$ represents the total output of industry j. Let ${\;A}_{\text{ij}}$ be the direct consumption coefficient:

(1) $$A_{\text{ij}}=\frac{\text{Xij}}{\text{Xj}}$$

$A=\left(a_{\text{ij}}\right)$ is the matrix of direct consumption coefficients, and the economic system β can be expressed as:

(2) $${X=\text{AX}+Y=\left(I-A\right)}^{-1}Y$$

In Equation (2), ${\;\left(I-A\right)}^{-1}$ represents the Leontief inverse matrix. Let $b_{\text{ij}}$ be the complete consumption coefficient, which means the value of direct and indirect consumption in industry i per unit change in final demand in industry j, and $B=\left(b_{\text{ij}}\right)$ be the complete consumption matrix, according to the input-output existence relationship:

(3) $${B=A\left(I-A\right)}^{-1}$$

The hypothetical extraction method commences by assuming that a specific industry is eliminated from the economic system. Subsequently, it assesses the correlation effect between industries by comparing the total social output prior to and subsequent to extraction and evaluates the significance of this industry within the economic system.

The entire economic system β can be viewed as consisting only of industry ${\;\beta }_{s}$ and industry $\beta_{-s}$, and ${\;\beta }_{i,j}$ denotes the input of industry sector i to industry sector j. Then, the economic system β can be expressed as:

(4) $$\beta =\left[\begin{array}{cc} \beta_{s,s} & \beta_{s,-s}\\ \beta_{-s,s} & \beta_{-s,-s} \end{array}\right]$$

According to Equations (3), (4), the economic system before industry $\beta_{s}$ extraction can be expressed as:

(5) $$\left[\begin{array}{c} x_{s}\\ x_{-s} \end{array}\right]=\left[\begin{array}{cc} A_{s,s} & A_{s,-s}\\ A_{-s,s} & A_{-s,-s} \end{array}\right]\times \left[\begin{array}{c} x_{s}\\ x_{-s} \end{array}\right]+\left[\begin{array}{c} Y_{s}\\ Y_{-s} \end{array}\right]=\left[\begin{array}{cc} \alpha_{s,s} & \alpha_{s,-s}\\ \alpha_{-s,s} & \alpha_{-s,-s} \end{array}\right]\times \left[\begin{array}{c} Y_{s}\\ Y_{-s} \end{array}\right]$$

where $\left[\begin{array}{cc} \alpha_{s,s} & \alpha_{s,-s}\\ \alpha_{-s,s} & \alpha_{-s,-s} \end{array}\right]={\left(I-A\right)}^{-1}$ is the Leontief inverse matrix.

After industry $\beta_{s}$ is extracted, the matrix form of the economic system of industry $\beta_{s\;}$, if no economic activity occurs with industry $\beta_{-s}$, can be expressed as:

(6) $$\left[\begin{array}{c} x_{\;s}^{\#}\\ x_{\;-s}^{\#} \end{array}\right]=\left[\begin{array}{cc} A_{s,s} & 0\\ 0 & A_{-s,-s} \end{array}\right]\left[\begin{array}{c} x_{\;s}^{1}\\ x_{\;-s}^{1} \end{array}\right]+\left[\begin{array}{c} Y_{s}\\ Y_{-s} \end{array}\right]=\left[\begin{array}{cc} {\left(I-A_{s,s}\right)}_{-1} & 0\\ 0 & {\left(I-A_{-s,-s}\right)}_{-1} \end{array}\right]\times \left[\begin{array}{c} Y_{s}\\ Y_{-s} \end{array}\right]$$

Then, Equations (5), (6) give the difference in total social output before and after extraction:

(7) $$X-X^{\#}=\left[\begin{array}{cc} X_{S} & {-X}_{\;S}^{1}\\ X_{-S} & {-X}_{\;-S}^{1} \end{array}\right]=\left[\begin{array}{cc} \alpha_{s,s}{-\left(I-A_{s,s}\right)}^{-1} & \alpha_{s,-s}\\ \alpha_{-s,s} & \alpha_{-s,s}{-\left(I-A_{-s,-s}\right)}^{-1} \end{array}\right]\times \left[\begin{array}{c} Y_{s}\\ Y_{-s} \end{array}\right]=\left[\begin{array}{cc} \varphi_{s,s} & \varphi_{s,-s}\\ \varphi_{-s,s} & \varphi_{-s,-s} \end{array}\right]\times \left[\begin{array}{c} Y_{s}\\ Y_{-s} \end{array}\right]$$

Based on Equation (7), assuming that **γ** is the unit vector, the total $\beta_{s\;}$, correlation can be expressed as:

(8) $${\text{TL}}_{s}=\gamma \left[\begin{array}{c} X- \end{array}X^{*}\right]$$

The backward correlation is ${\text{BL}}_{s}=\gamma \left[\varphi_{s,s}\;\varphi_{-s,s}\right]Y_{s}$, and the forward correlation is ${\text{FL}}_{s}=\gamma \;\left[\begin{array}{c} \varphi_{s,-s}\\ \varphi_{-s,-s} \end{array}\right]$, according to its economic implications: ${\text{TL}}_{s}={\text{BL}}_{s}+{\text{FL}}_{s}$.

### 3.3 Vertical integration of labor force population consumption

Here, we introduce the matrix representing the labor force population in China by industry IV, $\text{IV}={\left[{\text{iv}}_{1}\;\ldots {\text{iv}}_{i}\ldots {\text{iv}}_{n}\right]}^{\prime }$, ${\text{iv}}_{i}$ is the amount of labor force population in i industries, i.e., direct consumption.

Let P be the direct labor force population consumption coefficient and $P_{i}$ represent the direct labor force population consumption coefficient for sector i; then, according to equation (1), we obtain:

(9) $$P_{i}=\frac{{\text{iv}}_{\text{ij}}}{x_{j}}$$

According to Equations (8), (9), the vertically integrated consumption of the labor force population in sector j is:

(10) $${\text{VIC}}_{j}^{\#}=\sum_{i=1}^{n}P_{i}A_{\text{ij}}\;Y_{j}$$

In Equation (10), ${\text{VIC}}_{j}^{\#}$ denotes the labor force population directly and indirectly utilized by sector j to reach final demand, $A_{\text{ij}}$ is the Leonitef inverse matrix element, and $Y_{j}$ denotes the final demand of industry j. On the basis of the vertically integrated measurement method, Sanchez Cholis and Duarte improved the hypothetical extraction method by decomposing the sectoral labor force demographic linkage effects in the form of vertically integrated consumption The labor force demographic correlation effect of sectors is decomposed into four components: internal, composite, net antecedent and net consequent, and the labor force demographic correlation between industrial sectors of the economic system is measured through the change of labor force demographic transfer. By numerically representing the consumption characteristics of the industry, vertically integrated consumption enables a more precise analysis of the direct and indirect utilization of the labor force population in each sector by combining the direct and total consumption coefficients of the labor force population with the final demand.

### 3.4 Model of labor force demographic association effects by industry under MHEM

Currently, the HEM paradigm is widely utilized in industrial linkage studies across various fields [37]. There are three limitations of the HEM. The assumption is that the entire industrial sector has been completely extracted from the system, which eliminates only the direct effects, not the indirect ones. Furthermore, the interconnectedness among various sectors means that fully extracting one sector will impact the relationships with other sectors, resulting in alterations in the intermediate inputs of those sectors. This is especially accurate when the sector being removed is a crucial sector. In these situations, intermediate inputs from other sectors cannot stay consistent. If a sector is fully extracted, its products circulate exclusively within itself, severing all connections with other sectors, which is an implausible scenario. Duarte et al. proposed the Modified Hypothetical Extraction Method (MHEM) to overcome the limitations of HEM [47]. By integrating HEM with a longitudinal integration algorithm, the correlation effect can be classified into four factors: internal effect, composite effect, net forward transfer, and net backward transfer, allowing for a more precise and quantitative analysis.

A model of the demographic association effects of labor utilization by industry in China is constructed based on the MHEM article. MHEM combines the traditional HEM with VIC in the form of vertically integrated consumption decomposing equation into four independent association factors: Internal Effect (IE) indicates the utilization of labor force within $\beta_{s}$ industry as shown in Equation (11); Mixed Effect (ME) indicates the amount of labor force that is reinflated into $\beta_{s}$ industry after $\beta_{s}$ industry purchases $\beta_{s}$ industry products as intermediate inputs and used for final demand as shown in Equation (12); Net Backward Linkage (NBL) indicates the total labor force in $\beta_{-s}$ industry that is directly and indirectly utilized by $\beta_{-s}$ industry to meet final demand by purchasing $\beta_{-s}$ industry products as intermediate inputs ${\;\beta }_{s}$, the net backward transfer of labor force; Net Forward Linkage (NFL) indicates the labor force in $\beta_{-s}$ industry that is utilized by $\beta_{-s}$ industry products purchased by $\beta_{-s}$ industry as intermediate inputs $\beta_{s}$, the net backward transfer of labor force. Net forward linkage (NFL) indicates the size of the labor force population in industry $\beta_{-s}$ purchased by industry $\beta_{-s}$ for use as intermediate inputs, i.e., the net forward shift of the labor force population. IE, ME, NFL and NBL are:

(11) $$\text{IE}=P_{s}{\left(I-A_{s,s}\right)}^{-1}Y_{s}$$

(12) $$\text{ME}=P_{s}\left[\alpha_{s,s}{-\left(I-A_{s,s}\right)}^{-1}\right]Y_{s}$$

(13) $$\text{NFL}=P_{s}\alpha_{s,-s}Y_{-s}$$

(14) $$\text{NBL}=P_{-s}\alpha_{-s,s}Y_{s}$$

At the level of the entire economic system, the economic ramifications of vertically integrated consumption (VIC) and direct consumption (DC) indicate that the aggregate direct consumption of the labor force across all sectors is equivalent to the cumulative VIR consumption. Furthermore, the labor force's net forward and backward correlation effects result in an equal sum. According to the economic implications of the four correlation effect factors, direct consumption, and vertically integrated consumption: VIC=IE+ME+NBL, DC=IE+ME+NFL.

The net transfer of the labor force population (Net Transfer, NT) is obtained from Equations (13), (14):

(15) NT=NBL−NFL

The labor population of other industries is transferred to this industry if NT is greater than 0 as shown in Equation (15), and the labor population of this industry is transferred to other industries if NT is less than zero. The total labor population force consumed directly and indirectly by each sector to obtain the final demand is equal to the labor force utilized directly by each sector throughout the industrial chain. By way of the exchange of intermediate inputs, the quantity of laborers exported to other sectors is equivalent to the aggregate quantity of laborers imported from other sectors by each industry sector. The enhanced HEM method identifies the internal transfer flows of the labor population force within each sector in addition to quantifying the correlation effect of labor population force consumption between industrial sectors.

### 3.5 Distance of propagation of labor population force population transfer under structural path lengths

Utilizing the SPA methodology, it is possible to ascertain how, and through which sectors the labor population completes the transmission between entire sectors. Creating a comprehensive map of the interactions of labor force transfers between the transportation sector and the rest of the economy is possible by examining the inverse perspective of the labor population transmission path. The expansion of the Leontief inverse matrix is showed in Equation (16):

(16) $$L={\left(I-A\right)}^{-1}=I+A+A^{2}+A^{3}+\ldots +A^{t},\lim_{t\to \infty }\left(A^{t}\right)=0$$

(17) $$R=e{\left(I-A\right)}^{-1}Y=\underset{{\text{PL}}^{0}}{\underbrace{\text{eIY}}}+\underset{{\text{PL}}^{1}}{\underbrace{\text{eAY}}}+\underset{{\text{PL}}^{2}}{\underbrace{eA^{2}Y}}+\underset{{\text{PL}}^{3}}{\underbrace{eA^{3}Y}}+\ldots +\underset{{\text{PL}}^{t}}{\underbrace{eA^{t}Y}}=\left[\begin{array}{c} \begin{array}{c} e_{1}y_{1}\\ e_{2}y_{2}\\ \vdots \end{array}\\ e_{n}y_{n} \end{array}\right]+\left[\begin{array}{c} e_{1}\sum_{i=1}^{n}a_{1,i}y_{i}\\ e_{2}\sum_{i=1}^{n}a_{2,i}y_{i}\\ \vdots \\ e_{n}\sum_{i=1}^{n}a_{n,i}y_{i} \end{array}\right]+\left[\begin{array}{c} e_{1}\sum_{i,j=1}^{n}a_{1,j}a_{j,i}y_{i}\\ e_{2}\sum_{i,j=1}^{n}a_{2,j}a_{j,i}y_{i}\\ \vdots \\ e_{n}\sum_{i,j=1}^{n}a_{n,j}a_{j,i}y_{i} \end{array}\right]+\left[\begin{array}{c} e_{1}\sum_{i,j,k=1}^{n}a_{1,k}a_{k,j}a_{j}y_{i}\\ e_{2}\sum_{i,j,k=1}^{n}a_{2,k}a_{k,j}a_{j}y_{i}\\ \vdots \\ e_{n}\sum_{i,j,k=1}^{n}a_{n,k}a_{k,j}a_{j}y_{i} \end{array}\right]+\cdots$$

Equation (17) measures the increase in impact propagation along paths caused by non-linear factors. The quantification of the impact propagation along interconnected paths is determined by the existence of non-linear factors [48]. The average structural path step transfer propagation distance of the labor force population through path multipliers is determined by the average number of steps taken by a reverse demand pull or positive cost push in one sector to influence output in another sector, known as the average propagation distance (APL). Calculating the APL measure between path destination industry *i* and path origin industry *j* sectors *i* and *j* can be calculated as follows [15]:

(18) $$q_{\text{ij}}=\left\{ \begin{array}{c} \frac{h_{i\;j}}{l_{i\;j}},i\neq j\\ \frac{h_{i\;j}}{\left(l_{\text{ij}}-1\right)},i\neq j \end{array}\right.$$

where $q_{\text{ij}}$ denotes the elements of the Leontief inverse matrix obtained in (1) and $h_{\text{ij}}$ denotes the elements of the matrix H=L(L−1) as shown in Equation (18).

The closer $q_{\text{ij}}$ is to 1, the more prominent the direct dependence is compared to the indirect linkage between the two sectors. Applying the 2005–2020 input-output tables separately indicate the proximity of linkages between sectors. $q_{\text{ij}}$ greater than 3 indicates the predominance of direct or single-step mediated linkages at this level of aggregation.

### 3.6 Labor force population data

Current research primarily assesses this by using age categories [49]. Calculate the net inflow rate by determining the difference between the resident population and the household population as a proportion of the household population [50]. None of them provide a genuine and precise representation of the labor force's movement [51]. This paper utilizes the population in employment as a metric to assess labor force practices, employing the rate of change in the population in employment to indicate local labor mobility [52]. This paper will utilize the number of employed individuals in urban non-private sectors across 19 industries in China from 2005 to 2020 as the data for labor force mobility.

## 4 Empirical studies

### 4.1 Data sources and industry groupings

The compilation of domestic input-output tables does not occur on an annual basis. The article relies on data from all input-output tables published by the National Bureau of Statistics (National Bureau of Statistics, 2005–2020) for the years 2005–2020 (specifically, 2005, 2007, 2010, 2012, 2015, 2017, and 2018–2020) and the number of individuals employed by each industry in China's labor population force during those years. 19 major industry sector clusters comprise the Chinese input–output tables from 2005 to 2020, as per the National Economic Industry Classification (GB/T4754-2017) standard (Ministry of Civil Affairs of the People's Republic of China, 2017) the main sectors include agriculture, forestry, animal husbandry and fishery products and services (S1) mining (S2) manufacturing, including ago-food processing, food manufacturing, textiles, pharmaceuticals, metal products, etc.; (S3) electricity, heat, gas and water supply (S4) construction (S5) wholesale and retail trade (S6) transport, warehousing and postal services (S7) accommodation and catering (S8) information transmission, software and information technology services (S9) Finance (S10) Real Estate (S11) Rental and Business Services (S12) Scientific Research and Technology Services (S13) Water, Environment and Public Facilities Management (S14) Residential Services, Repair and Other Services (S15) Education (S16) Health Social Work (S17) Culture, Sports, and Recreation (S18) public administration, social security, and social organization industry (S19). To satisfy the assumed preconditions of a closed economic system, consumption, fixed capital formation, and net exports (exports - imports) are combined into the final demand column, i.e., the difference between imports and exports is included in net exports and incorporated into the vector of the final demand column, and the effects of exports and imports are canceled out.

### 4.2 Results and discussion

#### 4.2.1 Labor population force demographic coefficient

**Fig. 1:**
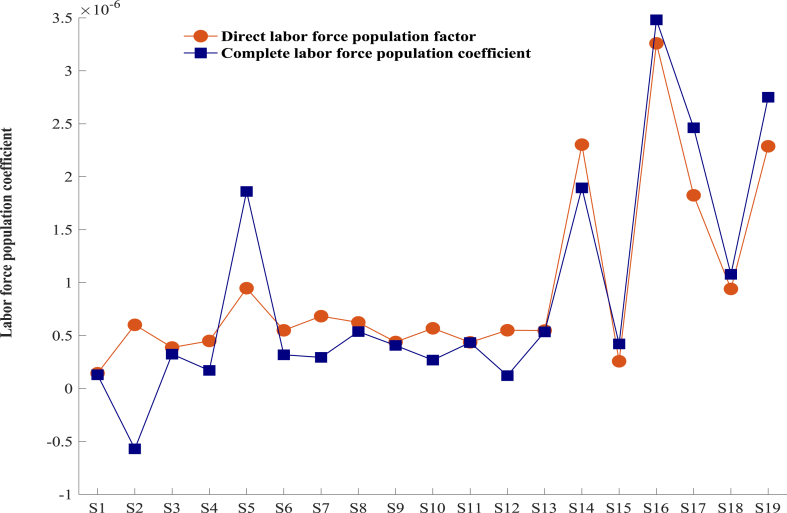
Direct labor population coefficients by industry sector in 2020.

The labor population force demographic coefficient (LFDC) represents the proportion of an industry sector's total output that corresponds to the size of its labor force. The labor population force in mining (S2), construction (S5), and transportation, warehousing, and postural services (S7) is higher than that of the other 16 industries, with construction (S5) being significantly larger than the other 18 industries, according to a comparison of the LFDC in 2020 (Fig. 1).

The industries with the smallest labor population coefficients are information transmission, software, and information technology services (S9), agriculture, forestry and fishery products and services (S1), manufacturing (S3). In 2020, agriculture, forestry, and fishery products and services will have the smallest labor population coefficient. Possible causes consist of the subsequent: Primarily, over an extended period, the overall volume and composition of China's residents' consumption exhibit substantial potential for growth, thereby establishing a favorable macroenvironment for the construction industry's progress in the medium and long term. Secondly, the integration of contemporary technology has resulted in enhanced commodity flow efficiency, thereby furnishing a robust assurance for the construction industry's progress. Lastly, the construction industry is an important pillar industry of the national economy, but also a labor-intensive industry amidst the severe conditions of the period. Inter-industry product transactions account for the indirect transportation of 33.8 %, 63.6 %, and 44.2 %, respectively, of the manufacturing, construction, transportation, warehousing, and postal industries with the largest direct labor force population volumes to the remaining industrial sectors. The remaining industrial sectors function as net importers of labor force, procuring products from other sectors to employ more than fifty percent of the labor force for final demand products. Agriculture and food manufacturing are the sectors that exert the greatest influence on labor force exports and imports within the broader economic system.

#### 4.2.2 Analysis of vertically integrated consumption versus direct labor population

Vertically integrated labor population force consumption reflects the direct and indirect labor population force consumption associated with a sector's final demand, and if the vertically integrated consumption of a sector's labor population force is greater than its direct consumption, it indicates that the production of the sector's products require the employment of labor population from other sectors of the overall economic system. Conversely, it indicates that the labor population force in that sector is transferred to other sectors of the economic system.

**Fig. 2:**
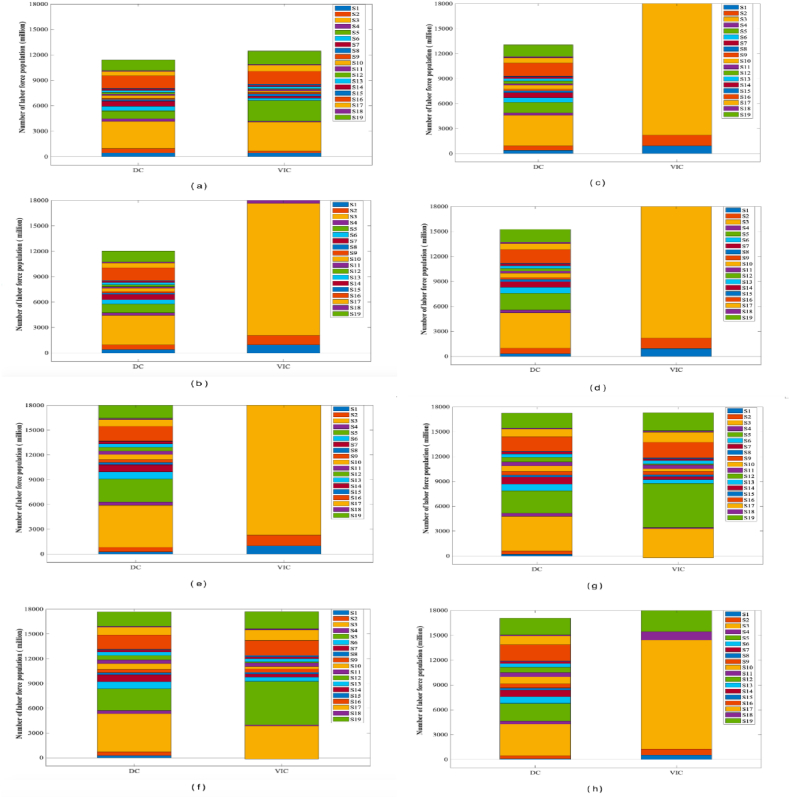
Labor population force population size and vertically integrated consumption by 2005–2020 (million) Note*.* (a) shows data for 2005. (b) shows data for 2007. (c) shows data for 2010. (d) shows data for 2012. (e) shows data for 2015. (f) shows data for 2017. (g) shows data for 2018. (h) shows data for 2020.

Based on the latest input-output table for 2005–2020 released by the National Bureau of Statistics of China and the data on the labor population employed in urban nonprivate units by industry for 2005–2020, by utilizing the most recent input–output table spanning the years 2005–2020, one can compute the direct consumption of the labor force in China by industry and the vertically integrated consumption of the labor population for the given time period (Fig. 2).

In Fig. 2(a), there is little difference in the share of each sector. The manufacturing industry's vertically integrated consumption in Fig. 2(b–d) represents the highest proportion of the total consumption of all 19 industries. This suggests that the manufacturing industry's vertically integrated consumption exhibited an upward trend from 2007 to 2010, and from 2012 onwards. Conversely, the manufacturing industry's vertically integrated consumption in Fig. 2(e–h) represents a declining proportion of the total consumption. From the standpoint of a single industry, the concept of vertically integrated consumption of labor population signifies that if the vertically integrated consumption of labor population exceeds the direct consumption population of that industry, then the industry's utilization of both direct and indirect labor populations to meet its final demand exceeds the actual utilized labor population as reported by the National Bureau of Statistics of Chi. The labor population force's net contribution to an industry is equal to the difference between VIC and DC. The value of DC minus VIC represents the labor population's net output to the industry, indicating that the sector functions as both a net exporter and a supplier of labor to the economy.

As seen from Table 1, industries whose vertically integrated consumption of the labor population force in 2020 is less than their direct consumption include mining, electricity, heat, gas and water supply, wholesale and retail, transport, storage and postal services, accommodation and catering, and other industries. All six of these industries are net exporters and suppliers of labor population forces to other sectors. The aggregate labor force utilized directly and indirectly by these industries is less than the actual labor force published by the National Bureau of Statistics of China. Likewise, the thirteen remaining industries receive labor populations from other industries and are net importers of labor populations. Vertically integrated labor population consumption is the highest among all industries, at 66.23 million, which is 0.86 times greater than their direct labor population consumption. With 50.904 million employees, manufacturing has the second largest vertically integrated consumption; this sector's vertically integrated consumption is 1.34 times that of direct consumption. The construction industry has the largest difference among 19 industries (35.229 million people) between direct consumption and vertically integrated consumption. This indicates that the construction industry receives the greatest number of workers from other industries, making it the largest net labor population input industry at 1.64 times its direct consumption. Furthermore, it demonstrates that the construction industry receives the largest amount of labor from other industries among all industries. The mining industry has the smallest difference between vertically integrated consumption and direct consumption of −8,068,000 and is also the largest net exporter of the labor population force population, indicating that the mining industry is the largest supplier of the labor population. The ratio of vertically integrated consumption to direct consumption of the labor population force in the mining industry is the smallest at −1.07, indicating that the total amount of labor population force directly and indirectly consumed by the mining industry to meet the final demand is much smaller than the actual number of labor population force as published by the NBS.

**Table 1:** China's labor force population by industry VIC and DC in 2020 (million).

Sector	VIC	Ranking	DC	Ranking	VIC-DC	Ranking	(VIC-DC)/DC	Ranking
S1	173.10	14	192.60	17	−19.40	9	−0.10	11
S2	−392.40	19	414.40	12	−806.80	19	−1.90	19
S3	3503.20	2	4178.30	1	−675.10	18	−0.20	13
S4	140.60	16	369.20	14	−228.60	13	−0.60	16
S5	5322.10	1	2710.90	2	2611.10	1	0.90	1
S6	477.40	5	823.30	6	−345.90	14	−0.40	14
S7	352.90	10	819.00	7	−466.10	17	−0.60	17
S8	232.30	12	269.80	15	−37.50	11	−0.10	10
S9	392.80	9	424.30	11	−31.50	10	−0.10	9
S10	330.50	11	699.30	8	−368.90	15	−0.50	15
S11	463.50	7	466.00	10	−2.50	7	−0.01	6
S12	117.20	18	529.50	9	−412.30	16	−0.8	18
S13	402.30	8	411.50	13	−9.20	8	−0.02	8
S14	214.40	13	260.60	16	−46.20	12	−0.18	12
S15	126.50	17	77.40	19	49.10	5	0.63	2
S16	1853.70	4	1735.60	4	118.10	4	0.07	7
S17	1231.20	5	912.40	5	318.80	3	0.35	3
S18	168.10	15	146.60	18	21.50	6	0.15	5
S19	2185.40	3	1817.50	3	367.90	2	0.20	4

**Fig. 3:**
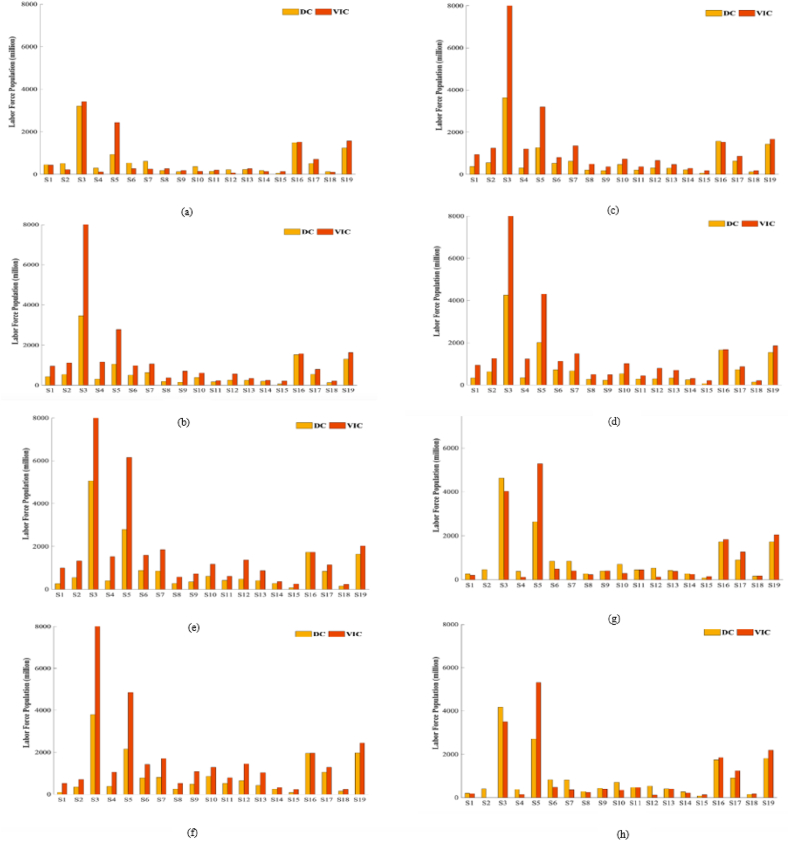
Direct and vertically integrated consumption of the labor population by 2005–2020 (million) *Note:*(a) shows data for 2005. (b) shows data for 2007. (c) shows data for 2010. (d) shows data for 2012. (e) shows data for 2015. (f) shows data for 2017. (g) shows data for 2018. (h) shows data for 2020.

Vertically integrated labor population force consumption reflects the direct and indirect consumption of labor population resources related to the final demand of a sector. As seen from Fig. 3(a–d), the manufacturing industry experiences a consistent annual increase in direct and vertically integrated labor population consumption. Fig. 3(e–h) demonstrates a decline in the indirect effect of manufacturing over the years. Notably, the construction industry exhibits a considerably higher direct and vertically integrated labor population consumption than the remaining 18 industries. However, starting from 2015, the manufacturing industry experiences a decline in such consumption. The sectors of manufacturing (38.06 million), construction (21.53 million), and transport, storage, and postal services (8.12 million), which have the highest direct labor population force in 2020, have a vertically integrated labor force consumption that is lower than their direct consumption and is transferred to the rest of the industry sectors through interindustry product trade. Interindustry product trading facilitated the indirect transfer and transportation of the labor population force of the remaining industrial sectors, accounting for 33.8 %, 63.6 %, and 44.2 % of their respective direct water consumption. In contrast, the mining industry (38.055 million), which has the largest direct labor population, consumes more of its labor force vertically than directly and receives an indirect transfer of 12.849 million people, or 33.8 % of its direct labor population, through interindustry trade of products. The vertically integrated consumption calculations revealed that only 24.7 % of the wholesale and retail direct labor population force was utilized for the labor force, while the remaining 75.3 % was net output; it was consumed directly and indirectly by the remaining sectors in the process of intermediate use. The wholesale and retail direct labor population force comprised 4.6 % of the total labor force in each sector. 7.28, 197, 195, 359, 94, and 12.97 million persons, respectively, comprised the net inputs.

The derivation demonstrates that, in accordance with the input-output model's significance, the multiplier of initial inputs to final demand is 1. The total vertically integrated consumption remains equivalent to the total amount of the direct labor population, irrespective of any modifications in the structure of final demand.

#### 4.2.3 Analysis of vertically integrated consumption components

**Fig. 4:**
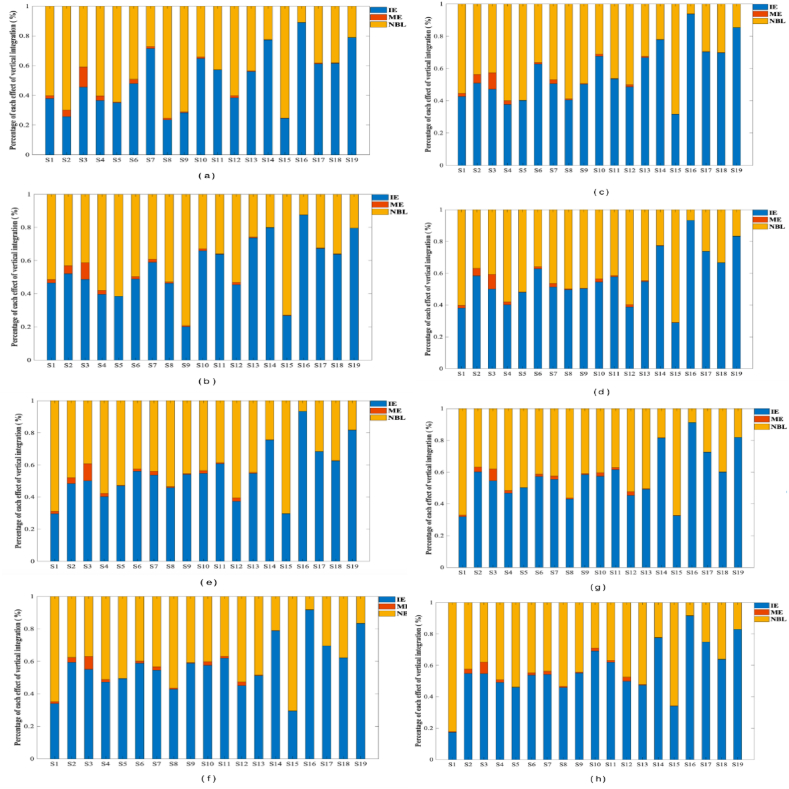
Composition of vertically integrated consumption by 2005–2020 *Note:*(a) shows data for 2005. (b) shows data for 2007. (c) shows data for 2010. (d) shows data for 2012. (e) shows data for 2015. (f) shows data for 2017. (g) shows data for 2018. (h) shows data for 2020.

Regarding the composition of the vertically integrated consumption of the labor population by industry, in 2015, the vertically integrated consumption of the labor population in the public administration, social security and social organizations industry (S19) was smaller than the direct consumption; in 2018 (S2) Mining (S3) Manufacturing industry, including the ago-food processing industry, the food manufacturing industry, the textile industry, the pharmaceutical industry, and the metal products industry, etc.; (S4) Electricity, heat, gas and water supply (S6) Wholesale and retail (S7) Transportation, storage and postal (S8) Accommodation and catering (S10) Finance, (S11) Real estate (S12) Leasing and business services, (S14) Water, environment and public facilities management, (S18) Culture, sports and recreation Labor population vertical integrated consumption is less than direct consumption; According to Fig. 1, Fig. 2 and the VIC equation, the composition of the vertically integrated consumption of the labor population force by industry in China from 2005 to 2020 can be Fig. 4.

Within the construction industry, the largest portion of its consumption is sourced from the labor population effect. This accounts for over 58.5 percent in each case, indicating that the majority of the labor force used by the construction industry comes from the wholesale and retail sector. The remaining 41.5 percent is obtained through purchased intermediate inputs, with 5.7 percent produced using labor population costs previously sold to other sectors. This means that a portion of the labor force involved in the acquisition of the final wholesale and retail sector comes from the wholesale and retail sector itself. Fig. 4(a–h) demonstrates that the labor force demographics in the construction industry remain stable, ranging from 0.4 to 0.5 throughout the year. Simultaneously, the demographic profile of the labor force in the construction sector is notable for its reliance on intermediate inputs, which are obtained from the labor force previously employed in other sectors. These inputs constitute the largest share of vertically integrated consumption (58.5 percent), indicating that a significant portion of the labor force in the construction sector is sourced from the transfer of intermediate inputs from other sectors.

The net backward linkages of all sectors, with the exception of mining, manufacturing, construction, accommodation and food services, account for more than fifty percent of their vertically integrated consumption. This indicates that the majority of the labor population required to produce the final demand products of these industry sectors is obtained indirectly through the procurement of intermediate inputs manufactured in other sectors. The employed labor force population is stable and the number of employed labor population resources is limited.

The mixed effect (ME) represents the amount of labor population force utilized by the β-s industry to purchase products of the β-s industry as intermediate inputs and then flow back to the β-s industry to be used in final demand. The net backward linkage (NBL) represents the amount of labor population force utilized directly and indirectly by the β-s industry to purchase products of the β-s industry to be used as intermediate inputs to satisfy final demand, i.e., the amount of labor population force utilized by the β-s industry, i.e., the amount of labor population force utilized by the β-s industry to satisfy final demand. Net backward linkage (NBL) represents the number of laborers in industry β directly and indirectly used by industry β to meet final demand by purchasing products from industry β as intermediate inputs, i.e., net backward transfer of labor population. Net forward linkage (NFL) represents the number of laborers in industry β used by industry β to meet final demand by purchasing products from industry β as intermediate inputs, i.e., net forward transfer of labor population.

According to the data in Table 2, the manufacturing sector has the largest workforce and also has the highest internal effect (25.45 million in manufacturing). This suggests that the labor force in this sector primarily relies on products produced within the sector itself, rather than on intermediate inputs purchased from outside the industry group. The substitution of these external inputs does not have a significant impact on the labor force in these sectors.

**Table 2:** Labor population correlation by economic sector 2020 (million).

SECTOR	DC	VIC	IE	ME	NBL	NFL	NFL-NBL
S1	192.60	173.10	55.30	1.67	116.12	135.60	19.53
S2	414.40	−392.38	−236.00	−11.84	−144.51	662.50	807.00
S3	4178.30	3503.20	1911.80	261.60	1329.80	2006.37	676.52
S4	369.20	140.56	65.70	2.28	72.56	301.40	228.80
S5	2710.90	5322.05	2666.80	5.60	2649.56	38.64	−2610.92
S6	823.30	477.36	273.76	6.48	197.12	543.30	346.20
S7	819.00	352.98	195.56	7.58	149.85	616.36	466.50
S8	269.80	232.30	99.90	1.50	130.80	168.61	37.74
S9	424.30	392.78	229.83	2.23	160.72	192.51	31.78
S10	699.30	330.47	189.67	7.39	133.41	503.62	370.22
S11	466.00	463.55	285.30	5.81	172.43	181.76	9.33
S12	529.50	117.23	53.16	2.71	61.36	474.46	413.09
S13	411.50	402.29	197.83	1.30	203.16	212.43	9.27
S14	260.60	214.42	174.70	0.35	39.36	85.63	46.27
S15	77.40	126.51	41.09	0.20	85.22	36.15	−49.06
S16	1735.60	1853.71	1688.93	0.79	163.98	69.57	−94.41
S17	912.40	1231.16	893.25	0.43	337.47	18.73	−318.75
S18	146.60	168.08	100.82	0.24	67.02	45.62	−21.40
S19	1817.50	2185.41	1789.90	0.90	394.61	26.78	−367.83
SUM	17258.20	17294.85	10677.42	297.24	6320.19	6320.19	0

#### 4.2.4 Labor population force population transfer by sector

**Fig. 5:**
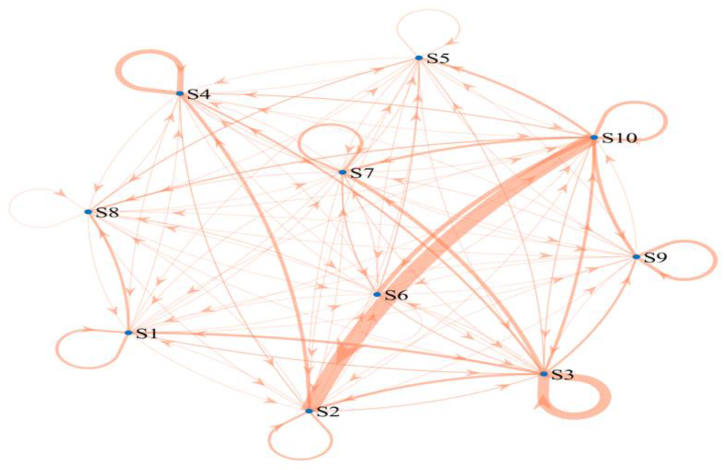
Labor population transfer paths and extent of transfer in 2020.

The framework for labor mobility across 19 industries, as depicted by the flow analysis, exhibited minimal changes between 2005 and 2020. Consequently, discerning alterations in observed flows from different years becomes challenging when examining the corresponding figures. For methodological and practical reasons, this article exclusively presents the path flow figures pertaining to the most recent year. According to Equation (14) and Equation (15) combined with Table 2, we can obtain the decomposition results of the labor population structural path transfer situation of China's 19 sectors in 2020, i.e., the labor population transfer paths and the degree of transfer in each sector in Fig. 5.

Labor population transfer influence is the full effect on the output of sector sectors *i* due to the transmission of an economic impulse form sector *j*. It can be demonstrated that global influence is the sum of the total influences along all the paths spanning two poles *i* and *j*. Furthermore, the structural paths can be utilized to quantify the direct and indirect impacts, thereby characterizing the connectivity paths of the network structure of the 19 sectoral economic systems. This is in addition to measuring the overall strength of upstream and downstream connectivity between sectors. The paper's analysis of transfer paths reveals 19 sectors that have significant transfer paths. Among the 19 sectors in 2020, mining and accommodation and food services, as well as leasing and business services, hold a central position. These sectors transfer a larger labor population compared to the other 17 sectors. Notably, the mining sector has the highest number of transfers with other sectors, indicating a clear disparity in the labor population compared to transfers within other sectors. The manufacturing sector exhibited the highest reliance on its own labor population in 2020, with the electricity, heat, gas, and water delivery sector following closely behind. The construction industry ranks first in terms of both nets backward correlation effects and net transfers in terms of labor population, which suggests that the construction industry is most dependent on other industries for its labor population in 2020.

#### 4.2.5 Average propagation lengths of the labor population by industry

**Fig. 6:**
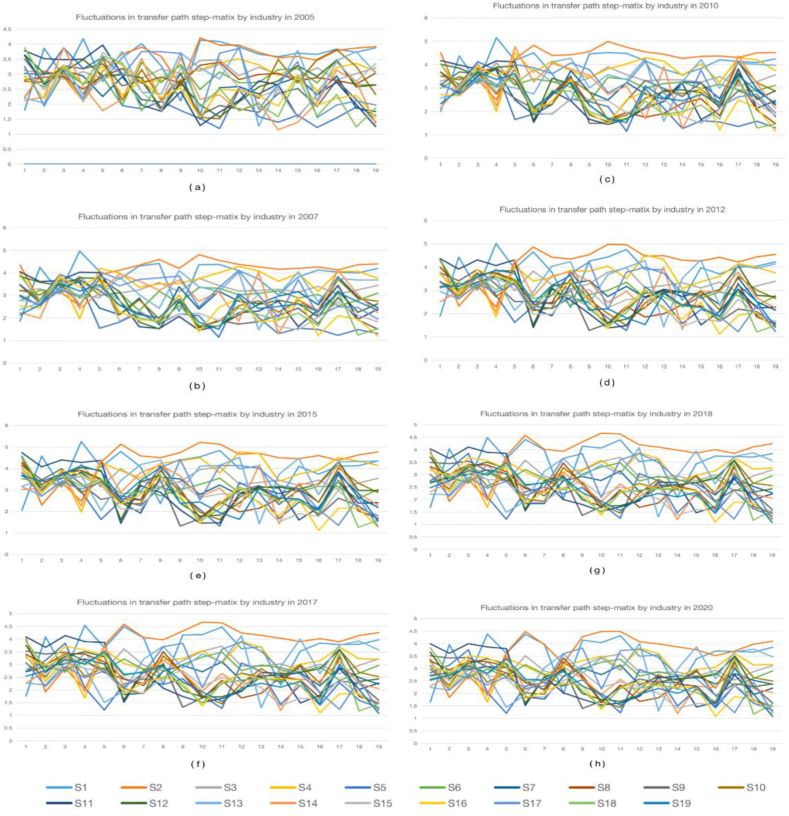
Path propagation distances for the APL in China's economic sectors 2005-2020 *Note:*(a) shows data for 2005. (b) shows data for 2007. (c) shows data for 2010. (d) shows data for 2012. (e) shows data for 2015. (f) shows data for 2017. (g) shows data for 2018. (h) shows data for 2020.

Based on the longitudinal analysis of Fig. 6, shows the results of computing the main paths of length 1 between the 19 aggregates of interest for this research alongside the measures of direct and global influence obtained by computing the matrices **AA** and **LL**. At a higher level of granularity, measures of the direct, indirect, and total influence between the digital and pharmaceutical sectors, with an emphasis on those influence paths having pharmaceuticals agriculture, forestry, and fishery products and services (S1) as the point of origin and as the point destination of economic impulse. It can be observed that the labor population transfer propagation distance from the water, environment, and public facilities management industry to the (S14) agriculture, forestry, and fishery products and services (S1), forestry and fishery products and services industry is the smallest compared to the other 17 industries, except for (S19) which has a distance value of 0.

This suggests that the transfer path for agriculture, forestry and fishery products and services is direct and short. In addition, the labor population transfer propagation distance from the (S10) water, environment, and public facilities management industry to the (S2) mining industry has the largest distance compared to the other 18 industries, indicating complex and lengthy transfer paths for agriculture, forestry and fishery products and services. Fig. 6(a) illustrates that the distance of propagation for the transfer of the labor force population is relatively small compared to other industries (S4) to (S9). Fig. 6(b–d) demonstrates that the labor population transfer propagation distance between the (S4) electricity, gas, water heat, and supply industry and the (S1) agriculture, forestry, and fishery products and services industry have the greatest propagation distance compared to the other 18 industries. This suggests that the transfer of agriculture, forestry, and fishery products and services from the electricity, gas, water heat and supply industry follow a significant and intricate path. Fig. 6(e) illustrates that (S15) has a shorter distance for the transfer of its own labor force population. Fig. 6(f) shows that the labor population transfer propagation distance of (S17) Health and Social Work to (S2) Mining is small relative to the other 18 industries, indicating that the transfer path of Agriculture, Forestry and Fisheries Products and Services is short and direct. Fig. 6(g) illustrates that the transfer of the labor force population spreads more directly from (S5) to (S6). Fig. 6(h) shows that the propagation distance of labor population transfer from (S10) Finance to (S2) Mining is large compared to the other 18 industries, which indicates that the transfer path of agriculture, forestry and fishery products and services is complex and influential.

## 5 Conclusions

After splitting the net forward and net backward correlation effects, the following conclusions were obtained:

Firstly, the manufacturing, construction, transport, storage, and postal services industries, which employ the largest number of workers, shift 33.8 %, 63.6 %, and 44.2 % of their workforce to other industrial sectors through interindustry product transactions. Except for mining, manufacturing, construction, accommodation and food services, and other industries, the remaining industry sectors have net backward linkages that contribute to over 50 percent of their vertically integrated consumption. This indicates that a significant portion of the labor force required to meet the final demand for products in these sectors is indirectly sourced through the purchase of intermediate inputs from other sectors.

Furthermore, the connection of labor force utilization across sectors refers to the movement of labor force demand between industries within the economic system. This rearrangement does not impact the overall labor force as long as the total final demand remains constant. The manufacturing sector, with the largest labor force, has the highest internal effect, with 25.45 million people employed in this sector, as well as the highest composite effect, with 3.15 million people employed in this sector.

Currently, China's inter-provincial industrial transfer is primarily driven by manufacturing industries, which aligns with the sector's significant vertically integrated consumption, compounding effects, and net forward correlation effects. Research on connections within the construction industry comprises the following: In a highly competitive global market, the manufacturing industry finds it challenging to gain a competitive edge on its own. Collaborative industries connected to both upstream and downstream sectors are more favorable for enhancing the industry's competitiveness [53]. In a competitive market, open synergies can enhance linkages between manufacturing and other industries to boost industrial development. The connections between manufacturing and other industries primarily involve a lower level of mutual support and have not advanced to a higher level of mutual reinforcement [53,54]. The findings from the literature align with the study in this paper, indicating that the manufacturing industry's labor force population has the most significant vertically integrated consumption and composite infectant net forward correlation effect. This implies that the manufacturing industry is highly self-correlated and has limited interactions with other industries. The construction industry plays a vital role in fostering economic growth across various sectors [55]. China's construction industry has a stronger ability to pull demand from other industries and push its own growth. This perspective aligns with the significant finding of this paper that the construction industry relies on the rest of the economic system to provide its workforce.

In summary, to better utilize the role of the labor population force in China's various industries, the article proposes the following recommendations:

First, the government should establish a human resource development system to promote employment, focus on how to enhance the employment skills and entrepreneurial qualities of workers, strengthen the ability of workers to adapt to new environments and external shocks, and strengthen the reserve of “stable employment” policies [41,[56], [57], [58], [59]]. Second, the government should conduct a comprehensive analysis of the issues that limit the employment of the labor population force from many angles. It should propose the creation of a unified labor market as the main solution for ensuring stable employment. Third, the government should strengthen the flexibility of the transfer of labor population demand in various industries, support employers docking training and be included in the employee reserve plan [[60], [61], [62]]. Fourth, the government should establish a labor population market policy conducive to the matching of supply and demand and reduce the pressure of unemployment in the community.

## Data availability statement

The data that support the findings of this study are available on request from the corresponding author.

## CRediT authorship contribution statement

**Xuan Li:** Conceptualization, Methodology, Resources, Data curation, Software, Formal analysis, Visualization, Writing – original draft, Writing – review & editing. **Yueyang Li:** Data curation, Methodology, Visualization, Writing – original draft, Writing – review & editing. **Yu Song:** Conceptualization, Supervision, Formal analysis, Methodology, Resources, Funding acquisition, Writing – original draft, Writing – review & editing.

## Declaration of competing interest

The authors declare that they have no known competing financial interests or personal relationships that could have appeared to influence the work reported in this paper.
